# The Interactive Effects of Pulsed Grazing Disturbance and Patch Size Vary among Wetland Arthropod Guilds

**DOI:** 10.1371/journal.pone.0076672

**Published:** 2013-10-08

**Authors:** Anna R. Armitage, Chuan-Kai Ho, Antonietta Quigg

**Affiliations:** 1 Department of Marine Biology, Texas A&M University at Galveston, Galveston, Texas, United States of America; 2 Institute of Ecology and Evolutionary Biology, National Taiwan University, Taipei, Taiwan; 3 Department of Oceanography, Texas A&M University, College Station, Texas, United States of America; James Cook University, Australia

## Abstract

Pulse disturbances and habitat patch size can determine community composition independently or in concert, and may be particularly influential on small spatial scales for organisms with low mobility. In a field experiment, we investigated whether the effects of a pulsed disturbance that simulated a grazing event varied with habitat patch size. We focused on the short-term responses of multiple co-occurring emergent salt marsh arthropods with differing levels of mobility and dispersal potential. As part of a marsh restoration project, two types of emergent marsh structures were created: small circular mounds (0.5 m diameter) separated by several meters of aquatic habitat, and larger, elongated terraces (>50 m long). Study plots (0.25 m^2^) were established on both structures; in a subset of plots, we simulated a pulsed grazing disturbance event by clipping the aboveground tissue of emergent plants, primarily *Spartina alterniflora*. At the end of the two-month recovery period, *Ischnodemus* (Hemiptera: Blissidae) density was over 50% lower in disturbed treatments within both large (terrace) and small (mound) patches. Predatory spider treatment responses were similar to *Ischnodemus* responses, suggesting a trophic relationship between those two arthropod groups. Alternatively, spiders may have been directly affected by the loss of shelter in the disturbed plots. *Prokelisia* (Homoptera: Delphacidae), which are generally more mobile than *Ischnodemus*, were not affected by disturbance treatment or by patch size, suggesting the potential for rapid recolonization following disturbance. Larval stem borers decreased by an order of magnitude in disturbed plots, but only in the large patches. In general, the disturbance effects of vegetation removal on arthropod density and community composition were stronger than patch size effects, and there were few interactions between pulsed disturbance and patch size. Rather, emergent marsh arthropod responses to disturbance and habitat area treatments were linked to the dispersal potential and mobility of each individual taxon.

## Introduction

Spatial and temporal heterogeneity are widely recognized as important determinants of community assembly e.g. [1]. Pulsed, unpredictable disturbances can be particularly influential drivers of ecosystem structure e.g. [2]. Pulse disturbances, defined as short-term, discrete physical or chemical disruption to a habitat [2], are frequently a result of episodic grazing events e.g. [3], [4], [5]. Community-level responses to grazing disturbances usually vary among sites and species. Moderate grazing can maintain heterogeneity within vegetation assemblages, which is generally considered an ecosystem benefit [6]. In other cases, grazing episodes can greatly reduce plant biomass [7] or completely denude areas [8], [9], which in turn decreases the abundance of associated fauna [10], [11].

The response of ecological communities to disturbances is, in part, affected by the structure of the surrounding landscape [12]. In particular, habitat patch size and proximity to other patches can influence community assembly at landscape scales and at smaller (micro-landscape) scales, depending on the species of interest [13], [14]. The ecological communities relevant at localized spatial scales are typically comprised of small organisms, many of which have relatively low mobility. For these organisms, habitat size is closely and inversely linked to predation risk [15] and species richness [16], [17]. Habitat size is also related to recovery from disturbance events at a variety of spatial scales [18]. Response to and recovery from grazing disturbances may vary with patch size, though most experimental work to quantify the interaction between pulsed grazing disturbances and patch size has focused on terrestrial systems e.g. [19].

The effects of patch size on community assembly have been well documented in subtidal and emergent habitats at the land-sea interface, such as coastal wetlands e.g. [18], [20], [21]. These habitats are also prone to a variety of grazing impacts, ranging from relatively minor or localized effects, to nearly complete removal of emergent vegetation [7], [8], [22]. Numerically, the most common grazers in many coastal marshes are a variety of specialized and generalist insects, but they tend to remove a relatively small amount of plant biomass [23]–[26]. Larger grazers, including a variety of mammals – horses, nutria, and boars, for example – can have strong but localized effects on plant communities, sometimes removing substantial amounts of biomass from areas of marsh [5], [11], [22], [27], [28]. Grazing by periwinkle snails has also been linked to some cases of emergent marsh dieback [7].

Secondary production, defined as heterotrophic biomass, can be an indicator of ecosystem responses to a variety of disturbances. Arthropods (e.g., insects, spiders, and their kin) associated with emergent vegetation are an important part of secondary production in coastal marshes. These arthropod groups have high but variable biomass linked to pulsed emergence events [29], [30] and have complex direct and indirect trophic links to other ecosystem components [31], [32]. Furthermore, variations in mobility and dispersal abilities among different arthropod groups can yield insight into the variety of mechanisms by which patch size and grazing disturbances can impact the ecosystem. However, there has been little experimental work to quantify the interaction between pulsed grazing disturbances and patch size on a spatial scale relevant to these arthropods.

Given the frequency and potential severity of pulsed grazing disturbances in coastal marshes, our objective was to determine if recovery from grazing disturbances is linked to habitat patch size among multiple co-occurring emergent marsh arthropods with differing levels of mobility and dispersal potential. Arthropods associated with emergent marsh vegetation are an ideal study group because they are a diverse group with species that vary in mobility (winged vs. non-winged) and trophic status (predators vs. herbivores). In a study timed to correspond with critical emergence and reproductive periods and at an appropriately small spatial scale, we investigated the recovery of marsh arthropod densities following a pulse disturbance in the form of vegetation removal that simulated severe grazing [7], [8], [11], [22], [27], [28]. We created these disturbances within small and large habitat patches in order to identify links between disturbance and patch size. We hypothesized that taxa with higher mobility (winged) and predatory species would recover from disturbances more quickly, particularly within larger patches of habitat.

## Methods

### Ethics Statement

This study took place on the grounds of the Lower Neches Wildlife Management Area, which is managed by the Texas Parks and Wildlife Department (TPWD). TPWD provided full access permissions to the study site. This study did not directly involve specimen collection, but the lead author (Armitage) is authorized to collect plants and insects under scientific collection permit SPR-0708-303 (exp. 7/31/14), issued by TPWD. The field studies did not involve any threatened or endangered species. No vertebrates were involved in this study.

### Study Site

**Figure 1:**
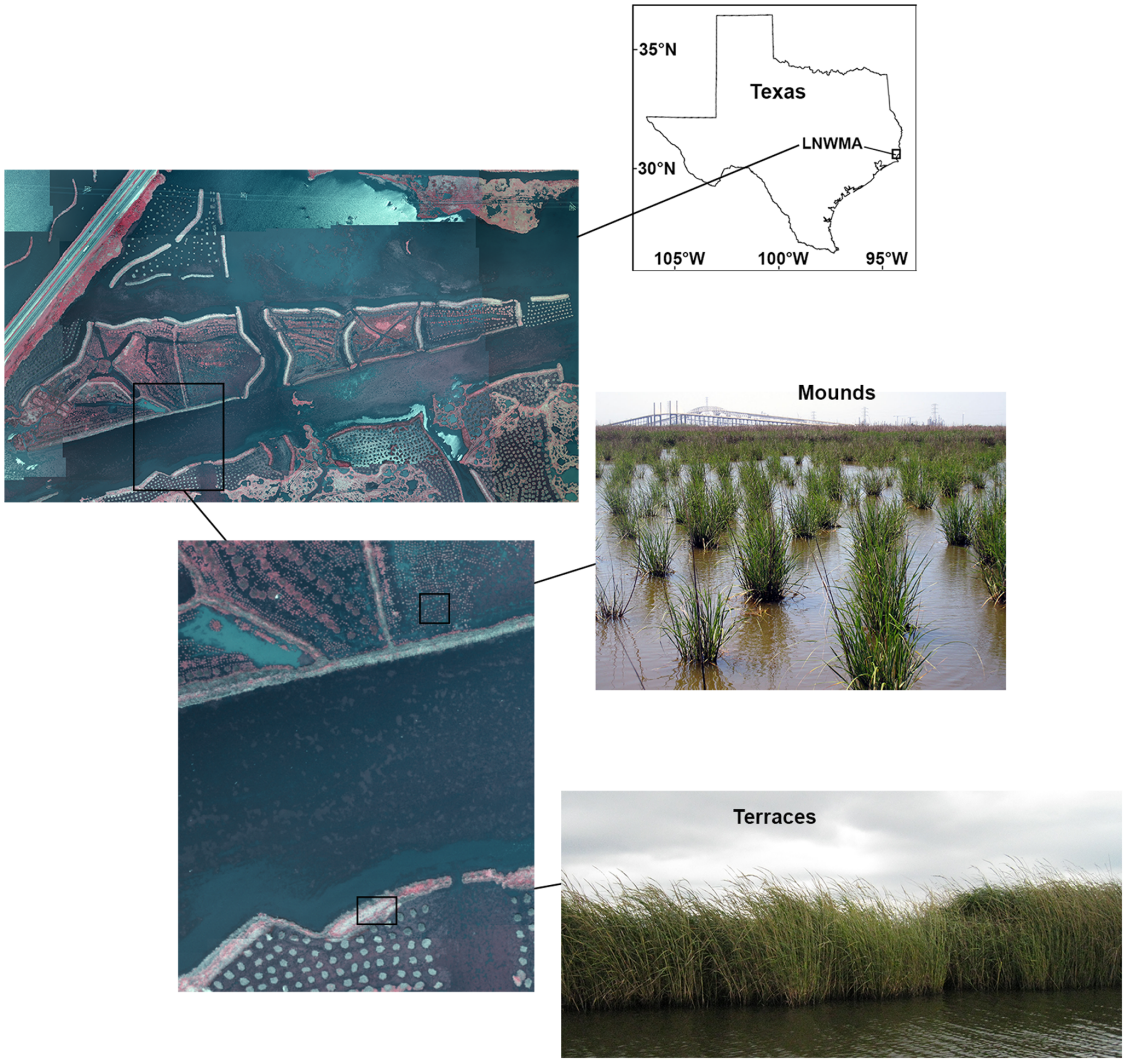
Map of study site. Infrared aerial images taken during a flyover of the marsh restoration project in the Lower Neches Wildlife Management Area (LNWMA) in September 2009, and ground-level pictures of terraces (large patches) and mounds (small patches).

Our study was conducted within the Lower Neches Wildlife Management Area (latitude 30° 0′ 5′′ N, longitude 93° 51′ 49′′ W) in Texas, USA. The study site initially consisted of 2.8 km^2^ of remnant emergent marsh and open water. As part of a larger marsh restoration project, two types of emergent marsh structures, mounds and terraces (Fig. 1A–D), were created in open water areas in spring 2008. Both structures were built with material excavated from adjacent bottom sediment, but they varied in plant canopy structure and size. Mounds had a low diversity plant canopy and were comprised of small circular mounds about 0.5 m in diameter, spaced 1–2 meters apart, and planted with smooth cordgrass, *Spartina alterniflora* cv. Vermilion. Terraces had a somewhat more diverse plant canopy structure and were larger in area; they comprised of narrow bands of marsh up to 5 m in width and over 200 m in length, and were planted with *Spartina alterniflora* cv. Vermilion and *Schoenoplectus californicus*. Species will be referred to generically hereafter. Mounds and terraces were spaced ∼100 m apart, but experienced very similar tidal influence. Ongoing monitoring of the restoration project verified that the soil and water characteristics were similar between habitat types (A.R. Armitage, unpublished data).

### Field Experiment

During the 2009 growing season (April – September), we conducted field experiments to investigate how patch size and disturbance influenced the arthropod community associated with the emergent marsh vegetation. We established 24 0.25-m^2^ study plots at the study site. Plots were at least 3 m apart. Half of the plots were on mounds, which were considered to be small patches. The mounds were 0.5 m in diameter, so a single study plot encompassed nearly an entire mound and was surrounded by aquatic habitat. The remaining plots were on terraces and were primarily in *Spartina* stands; these plots were placed within a larger stand of emergent vegetation and were therefore considered to be within large patches of habitat. In a subset of plots on both mounds and terraces, we created a one-time physical disturbance at the beginning of experiment. For the disturbance treatment, we clipped the emergent parts of all plants, removed all clipped plant material, and left the submerged parts of the stems (about 10 cm long) alone. This type of disturbance resembled patchy damage from pulsed herbivory events. In our study area, nutria (*Myocastor coypus*) are the most common herbivores that can potentially cause partial or complete leaf and stem removal C.-K. Ho pers. obs.[5], [28], [33]–[37]. The remaining plots remained unclipped as controls (n = 6 for each treatment).

At the end of the growing season in August/September 2009, we surveyed plant responses to experimental treatments. In each experimental plot, we took a photo and determined total plant coverage with VegMeasure v1.6, a spectral identification program. We also recorded the number of plant species present, and stem density and plant height for each plant species. For stem density, we counted all *Schoenoplectus* stems within each plot. Due to high *Spartina* density, we subsampled 10 cm×10 cm subsections of each plot for *Spartina* stem density. *Spartina* was the dominant species in our terrace plots, typically comprising 90–100% of plant coverage, and was the only plant species present on the mounds. Therefore, we performed additional measurements of *Spartina* fitness by haphazardly selecting three *Spartina* plants per plot to record leaf chlorophyll *a* content with a Konica Minolta SPAD-502 chlorophyll meter. For plant metrics where three sub-replicates were measured within an experimental plot, data were averaged to produce a single value for that plot, and plot averages served as replicates.

Arthropods were surveyed monthly throughout the growing season (April through September 2009). On each of the monthly surveys, we used a standard entomological visual counting technique to tally the number of herbivorous and predatory arthropods on three randomly selected *Spartina* plants [23], [26], [38]. An experienced practitioner identified arthropods to the lowest practical identification level in the field in order to minimize disturbance of any trophic interactions. In most cases, we used arthropod density, reported as number per 100 cm linear height of *Spartina* stem in order to standardize counts to plant size. The frequency of stem borers, which are primarily larval arthropods that burrow into plant tissue, was reported as the percentage of stems in the study plot with evidence of borer use at the end of the study period. The dominant herbivores included planthoppers *Prokelisia* spp., blissids *Ischnodemus conicus*, and stem borers; the most common predators were spiders (including lycosid and non-lycosid genera). All of these species typically are closely associated with *Spartina* plants and rely on their cryptic nature for protection; therefore, these arthropods exhibited a minimal flight response during our surveys. Our arthropod surveys on *Spartina* plants likely reflected the composition of the arthropod community in the study area because *Spartina* was the only plant species on mounds and the dominant one (typically ≥90% of plant coverage) on terraces. We rarely observed arthropods on *Schoenoplectus* plants on terraces, and those few arthropod species on *Schoenoplectus* were also typically found on *Spartina*.

### Data Analysis

Plant metrics and stem borer frequency were analyzed with two-way Analysis of Variance (ANOVA), where the independent variables were habitat type (small mounds, large terraces) and disturbance (control, disturbed). Homogeneity of variances was confirmed with Levene’s test of equality; stem borer data were log transformed accordingly.

Standardized densities for the three other most common arthropod groups (*Ischnodemus*, *Prokelisia*, and spiders) were analyzed with two-way repeated measures ANOVA, where the independent variables were habitat type and disturbance as above, repeated sequentially at 0.5, 1, 2, 3, and 4 months following the disturbance. Data were tested for normality using Mauchly’s test of sphericity; if significant, the Greenhouse-Geisser correction was applied to the analysis.

To examine the similarity of insect assemblages among disturbance treatments and habitat types, we summed the standardized densities of all arthropods over the time period, with the exception of stem borers, which were not reported as a density, but rather as a frequency of infected stems. Arthropod densities were very dynamic over time, so the accumulated densities calculation integrated treatment responses over time. We used PRIMER v6 (PRIMER-E Ltd., Plymouth Marine Laboratory, United Kingdom) to create a Bray-Curtis similarity matrix among treatments. We then used two-way analysis of similarity (ANOSIM) to identify differences in insect community composition among treatments. We used NMDS (nonmetric multidimensional scaling) ordination to represent these dissimilarities in Euclidean two-dimensional space.

## Results

**Figure 2:**
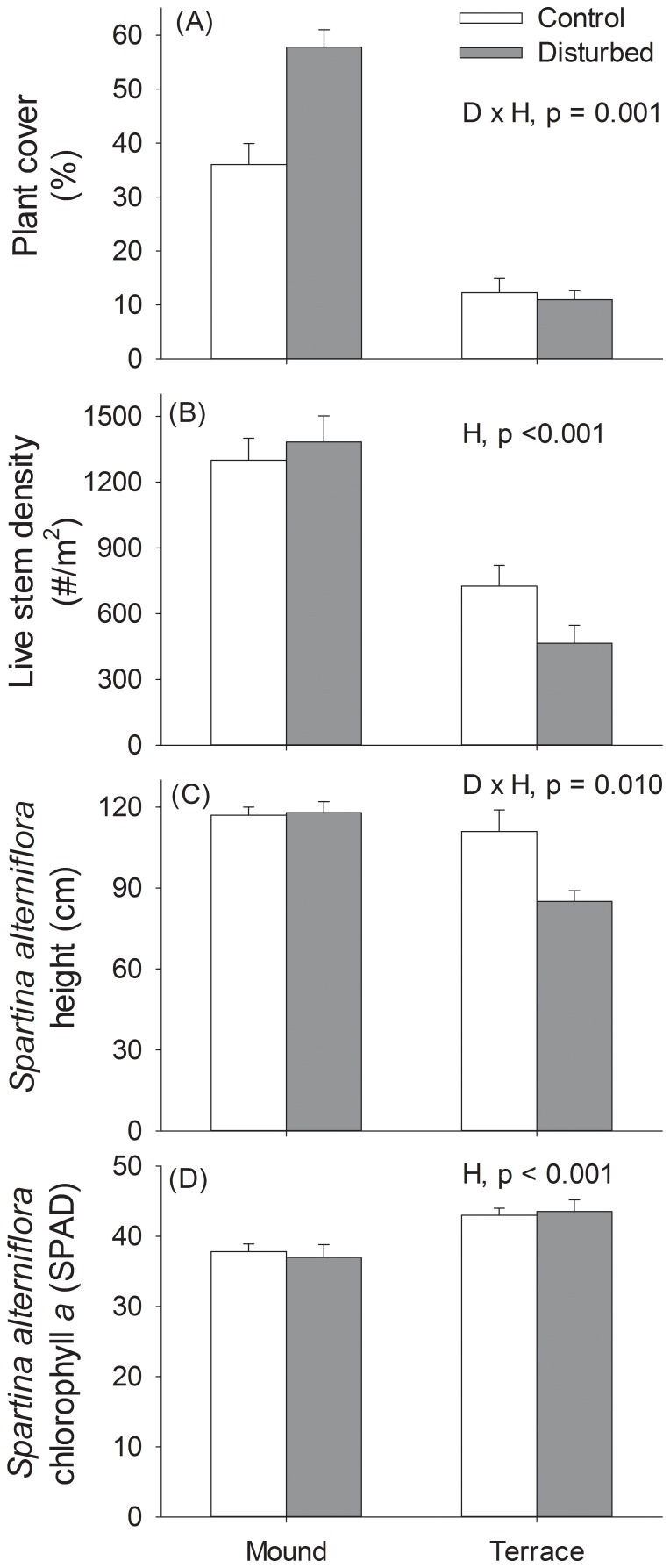
Disturbance and patch size effects on plant characteristics. Effects of disturbance treatments in large (terraces) and small (mounds) patches on plant characteristics: a) total plant cover, b) total live stem density, c) *Spartina alterniflora* stem height, d) relative *Spartina alterniflora* chlorophyll *a* content. Error bars represent standard error. Significant treatment effects from two-way ANOVA are noted; D = Disturbance, H = Habitat patch size.

By the end of the study period, treatment effects on plants were mostly small in magnitude, indicating that our plant removal treatment effectively created a pulsed disturbance. A significant interaction between disturbance and habitat type was driven by higher plant cover in disturbed plots on mounds (Fig. 2, Table 1). Plant cover was 80% lower on terraces than on mounds. Stem density was 50% lower on terraces than on mounds but was not affected by disturbance treatment. For plant height, there was a significant interaction between disturbance and habitat type because *Spartina* was about 20% shorter in disturbance treatments relative to controls on terraces but not on mounds. *Spartina* chlorophyll *a* concentration was slightly but significantly higher on terraces than on mounds, but there was no disturbance treatment effect (Fig. 2, Table 1).

**Table 1:** Summary of two-way ANOVA results for plant and stem borer responses to plant removal disturbance treatments in mound (small patch) and terrace (large patch) habitats.

Response variable	Factor	df	Mean square	F-value	p
Total plant cover	Disturbance	1	628.7	11.7	0.003
	Habitat	1	7441.6	138.5	<0.001
	Disturbance × Habitat	1	797.7	14.8	0.001
	Error	20	53.7		
Live stem density	Disturbance	1	47526.0	0.791	0.384
	Habitat	1	3342080.7	55.6	<0.001
	Disturbance × Habitat	1	178192.7	3.0	0.100
	Error	20	60056.9		
*Spartina* height	Disturbance	1	929.2	6.4	0.020
	Habitat	1	2217.0	15.3	0.001
	Disturbance × Habitat	1	1185.3	8.2	0.010
	Error	20	145.2		
*Spartina* leaf chlorophyll	Disturbance	1	0.08	<0.1	0.935
	Habitat	1	206.5	17.2	<0.001
	Disturbance × Habitat	1	2.4	0.2	0.659
	Error	20	12.0		
Stem borer frequency^a^	Disturbance	1	0.1	25.3	<0.001
	Habitat	1	0.1	39.3	<0.001
	Disturbance × Habitat	1	0.1	31.0	<0.001
	Error	20	<0.1		

a Stem borer p-values follow log transformation of the data.

**Figure 3:**
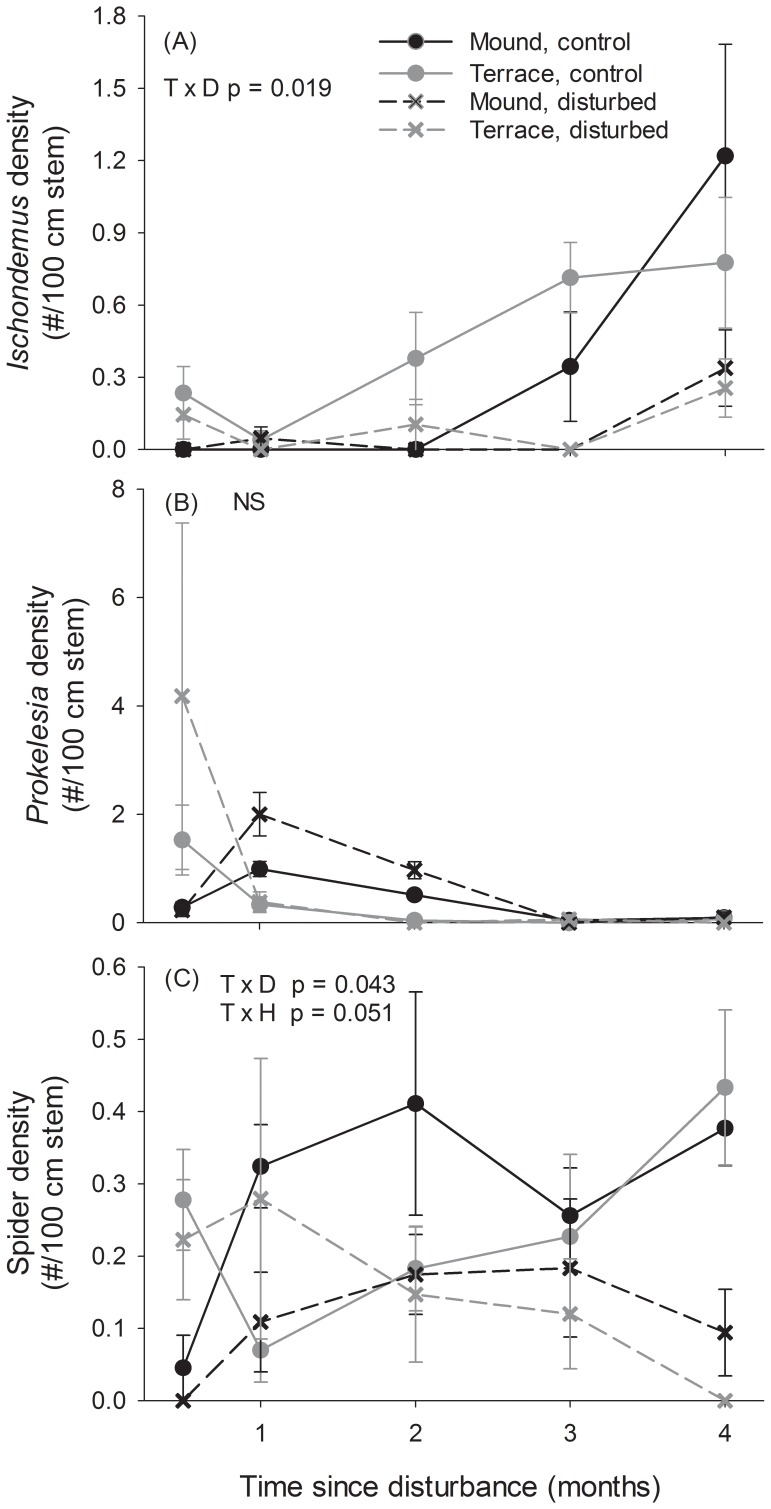
Disturbance and patch size effects on arthropod characteristics. Effects of disturbance treatments in large (terraces) and small (mounds) patches over time on arthropod characteristics: a) *Ischnodemus* density, b) *Prokelesia* density, c) spider density. Error bars represent standard error. Significant treatment effects from two-way repeated measures ANOVA are noted; T = time, D = Disturbance, H = Habitat patch size, NS = no significant treatment effects.

*Ischnodemus* (blissid) density varied over time and among disturbance treatments (time*disturbance p = 0.019; Table 2). At the end of the study period, *Ischnodemus* was up to 75% lower in plant removal treatments on both mound and terrace habitats (Fig. 3).

**Table 2:** Summary of two-way repeated-measures ANOVA results for arthropod densities in response to plant removal disturbance treatments in mound (small patch) and terrace (large patch) habitats over time.

Response variable	Factor	df	Mean square	F-value	p
*Ischnodemus*	Time	1.9	3.2	11.4	<0.001
	Time × Disturbance	1.9	1.3	4.5	0.019
	Time × Habitat	1.9	0.5	2.0	0.154
	Time × Disturbance × Habitat	1.9	0.3	0.9	0.408
	Error (Time)	37.4	0.3		
*Prokelesia* a	Time	1.05	38.5	3.0	0.095
	Time × Disturbance	1.05	6.9	0.5	0.477
	Time × Habitat	1.05	48.6	3.8	0.062
	Time × Disturbance × Habitat	1.05	11.7	0.9	0.351
	Error (Time)	20.9	12.7		
Spiders^a^	Time	4	<0.1	0.7	0.559
	Time × Disturbance	4	0.1	2.6	0.043
	Time × Habitat	4	0.1	2.5	0.051
	Time × Disturbance × Habitat	4	0.1	1.8	0.143
	Error (Time)	80	<0.1		

a p-values for *Prokelesia* and *Ischnodemus* follow the Greenhouse-Geisser correction.

*Prokelisia* (planthopper) density was not significantly affected by disturbance or habitat type, and did not vary over time, although there tended to be more planthoppers at the beginning of the study period in disturbed plots (Fig. 3, Table 2).

Spider density response to plant removal treatments and habitat type varied over time (Table 2). At the end of the study period, spider density was over 75% lower in plant removal treatments relative to controls in both habitat types (time*disturbance p = 0.043; Fig. 3). Spider densities on both mounds and terraces varied over time (time * habitat type p = 0.051; Fig. 3), but these temporal variations linked to patch size were stochastic.

**Figure 4:**
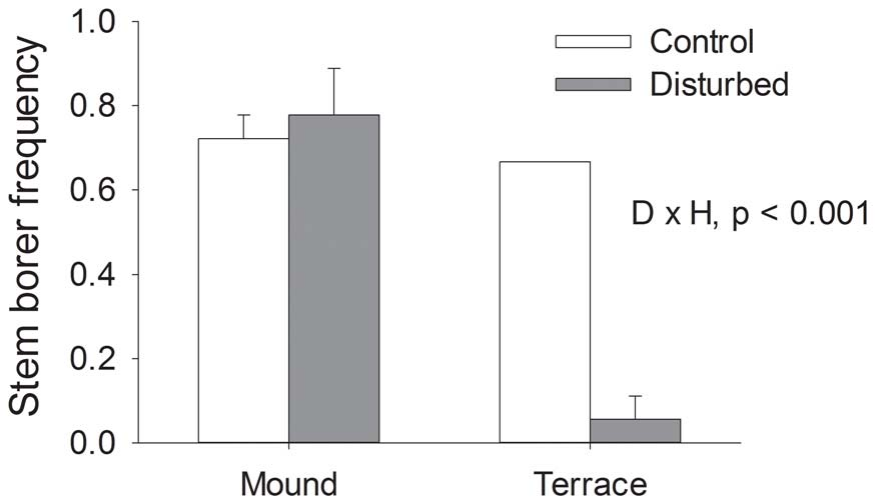
Disturbance and patch size effects on stem borers. Effects of disturbance treatments in large (terraces) and small (mounds) patches on stem borer frequency. Error bars represent standard error. Significant treatment effects from two-way ANOVA are noted; D = Disturbance, H = Habitat patch size.

There was a significant interaction between disturbance treatment and habitat type (p<0.001) on the frequency of stem borers at the end of the study. Disturbance reduced stem borer frequency by nearly an order of magnitude on terraces but not on mounds (Fig. 4, Table 1).

All other insects observed in the treatment plots occurred at very low densities. Insects in Family Miridae (*Trigonotylus uhleri*) were found in two of the 12 study plots on mounds and not at all on terraces; accumulated densities over the entire study period averaged less than 0.2/100 cm plant height. Ladybugs (Family Coccinellidae) were found in four treatment plots on mounds and not at all on terraces; accumulated densities averaged less than 0.3/100 cm plant height.

**Figure 5:**
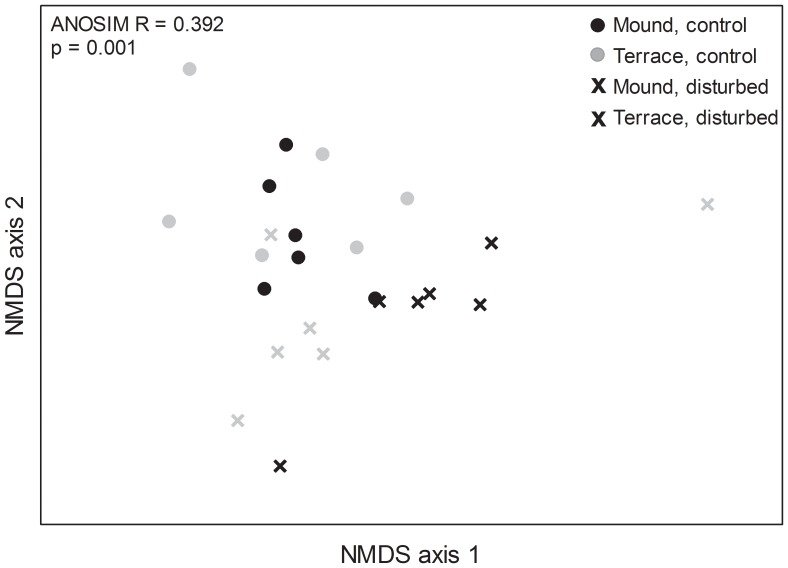
Disturbance and patch size effects on arthropod community composition. Composition of emergent marsh arthropod assemblages in response to disturbance treatment (disturbed, control) and habitat patch size (mounds, terraces). The nonmetric multidimensional scaling (NMDS) ordination is a representation of dissimilarities among treatments based on a Bray-Curtis similarity matrix. Results from ANOSIM are shown.

ANOSIM analyses generate an R statistic that indicates the degree of overlap among communities [39]. Communities in disturbed and control treatments were significantly different from each other (ANOSIM, p = 0.001), but the R statistic was relatively low (R = 0.392), suggesting that there was a fair amount of overlap between communities. The disturbed and control treatments were distinct in the MDS plot (2-dimensional stress = 0.1; Fig. 5). There was no significant difference in arthropod community composition between terrace (large) and mound (small) formations (R = 0.121, p = 0.076).

## Discussion

Our investigation is the first to our knowledge that evaluated the interactions between disturbance and patch size on fauna in coastal marshes, particularly with a focus on multiple co-occurring arthropod species with varying dispersal abilities and degrees of mobility. We effectively created short-term pulse disturbances, since plant removal had few significant negative or large effects on plant stem density, cover, or fitness. Disturbance effects on arthropod density and community composition were generally stronger than patch size effects. Only one group of arthropods, the larval stem borers, showed an interaction between disturbance and patch size treatments. Previous work on arthropods in wetlands and other grasslands suggests that patch size is an important driver of community assembly [16], [21], [40]. Our results differed from these studies by suggesting that the effects of physical disturbance (plant removal) were not necessarily stronger in the smaller patches (mound habitat). Rather, there was a community-level shift in species composition in response to plant removal disturbance, but not in response to patch size. Furthermore, the link between disturbance and patch size varied among taxa, and the treatment effects were linked to the dispersal potential and mobility of each individual species. It is not uncommon for disturbances to elicit species-specific responses [25], [32], [41]–[43], so we will discuss the treatment effects for each common taxonomic group.

*Ischnodemus* is a genus of herbivorous insects (Hemiptera: Blissidae) that can be found globally on a variety of host plants [44]. The blissid *Ischnodemus conicus* in this study typically uses *Spartina alterniflora* as its host species and often lives cryptically in basal leaf sheaths [44] C.-K. Ho, personal observation]. All *Ischnodemus* observed in our study site were micropterous (with reduced wings) and therefore had limited mobility. The close trophic link to its host plants and limited mobility may have constrained the distribution of *Ischnodemus* and made this species susceptible to environmental disturbance. Accordingly, *Ischnodemus* density was up to 75% lower in response to physical plant removal in both large (terrace) and small (mound) habitat sizes. There was a site-level increase in *Ischnodemus* abundance during the study period, but disturbed plots maintained lower densities than controls, regardless of habitat type. Limited adult mobility could constrain re-colonization following physical disturbances. Another mechanism of disturbed habitat colonization is the recruitment of newly emerged juveniles, suggesting that the disturbance treatment removed the cryptic larvae or eggs of this species. Although some species of *Ischnodemus* can produce multiple generations a year [45], the reproductive behaviors of the species in Texas are unknown, and it is possible that our study immediately followed a period of reproduction. In general, hemipterans are slow to recolonize following disturbance or isolation [46], [47], and the *Ischnodemus* in our study followed that pattern.

*Prokelisia* planthoppers (Homoptera: Delphacidae) in salt marshes occur in winged and non-winged forms, both of which specialize on the sap of *Spartina alterniflora* [43], [48]. Given this close trophic link, we expected that *Prokelisia* density would be closely related to *Spartina* canopy characteristics. *Prokelisia* was not affected by physical disturbance in either mounds or terraces, suggesting rapid recolonization as the cut plants grew back in. The proportion of winged individuals at our study site is unknown, but wing formation is sensitive to habitat disturbance [48], [49]. Therefore, winged forms may have occurred in our disturbed study area, facilitating colonization as the plants recovered from the physical disturbance. Furthermore, *Prokelisia* populations have a rapid turnover rate [50] and can be relatively mobile [51], suggesting that they have a high recolonization potential following disturbance.

“Stem borer” is a term applied to a wide variety of larval arthropods that burrow into plant tissue. In salt marshes on the Gulf Coast, they are most often comprised of lepidopterans [32], [52], [53]. They cannot be identified to species in the field without severe damage to the host plant, so we grouped all stem borers into one functional group. Stem borers remain in their excavated burrows until emergence; this close association suggests that stem borers will be sensitive to plant removal. We expected that stem borers would recover slowly following physical plant removal from the experimental plots, and that recovery would be slower in the smaller patch sizes (mounds). However, plant removal decreased stem borer frequency only in the larger terrace habitat. Since larval stem borers do not move between host plants and they are not reproductive, this surprising pattern may have been driven by a stochastic, small scale reproduction event by a group of adult lepidopterans on mounds. Alternatively, the stem borers may have in fact been postlarval insects such as beetles or flies that actively selected the mound habitat because of its higher *Spartina* coverage (Fig. 2). Furthermore, competition among different species of stem borers [52] may have muted disturbance responses on mounds.

Spider density was relatively low and variable over time, but all taxa observed, including hunting and web-building spiders, were predatory, so we grouped all spiders into a single functional group. Other predators such as omnivorous grasshoppers [54] were not observed in our study plots, and ladybugs (Family Coccinellidae) were uncommon. In both small (mound) and large (terrace) patches, spider density may have been linked to prey densities: where the experimental treatments affected prey, spiders responded similarly. Four months after plant removal, both *Ischnodemus* and spider densities were lower on mounds and terraces, suggesting a bottom-up trophic cascade that was independent of patch size. Similar bottom-up effects of prey availability on spiders have been documented in habitats ranging from salt marshes [55] to tropical forests [56]. Alternatively, the decrease in spider density in disturbed treatments may have been driven by the loss of shelter in the disturbed plots [57].

Our examination of treatment responses has thus far considered species individually or in terms of basic consumptive trophic relationships. However, emergent marsh arthropod communities often exhibit complex interactions within and among trophic levels. Stem borers, for example, can decrease planthopper densities by directly damaging planthopper eggs while burrowing into plant tissue, or by making plant tissue less attractive to planthopper adults [32], [58]. Likewise, intraguild predation can reduce top-down pressure on prey items [59]. Our study design was not intended to elucidate the nature of these indirect, multi-trophic interactions, though we assert that spider densities were relatively low, likely minimizing the influence of complex multitrophic interactions on arthropod community structure. Our intention was to explore the interactions between a pulsed grazing disturbance event and habitat patch size. We clearly documented that disturbance influenced emergent marsh arthropod community composition, and in one species group, the recovery from disturbance varied with patch size.

We expected to detect interactions between the pulsed grazing disturbance treatment and habitat patch size on arthropod density. Rather, recovery from pulse disturbance was similar across habitat types for most species, with the exception of the stem borers. Other types of disturbances, such as nutrient pulses, may have had longer-term effects on arthropod assemblages [43]. Furthermore, both small and large patches were embedded within a wetland restoration site, which is itself a form of disturbance, since the emergent marsh structures (mounds and terraces) used in this study had been created in an area that was previously open water. Among control plots, there were essentially no differences between patch sizes in terms of arthropod density. Insect assemblages can be sensitive to isolation from source populations [60]. The similar assemblages in both restored habitat types suggests that mounds and terraces were equally isolated from the reference area, which was presumably the source of new arthropod recruits to the entire restored area. Early colonizers of restored habitats tend to be more mobile taxa [46], [61], and even flightless insects can often travel several meters [62]. Therefore, the arthropods we encountered in our study plots after our disturbance treatments may have all had relatively high mobility at the spatial scale of our study plots, which could have muted interactions between disturbance and patch size treatments.

Arthropod communities associated with emergent vegetation in coastal marshes can be considered a “secret garden” of secondary productivity. They have high but variable biomass linked to pulsed emergence events [29], [30] and many complex direct and indirect trophic links to other ecosystem components [31], [32]. These links to other ecosystem components emphasize the importance of understanding arthropod responses to disturbances and patch size. Furthermore, treatment effects were closely linked to the dispersal potential and mobility of each individual arthropod species group, providing a broad perspective of the ecological dynamics of interactions between disturbance and patch size across a range mobility levels. Although the spatial scale of our disturbances was small, it was on a spatial scale that was relevant to our study organisms, and thus provides tools to make predictions about other ecosystem responses to co-occurrences of pulsed disturbance and patch size.
